# First Principles Study of Electronic and Optical Properties of Al‐P Co‐Doped ZnO in the Presence of Zn Vacancies

**DOI:** 10.1002/open.202400222

**Published:** 2024-10-17

**Authors:** Zhengguang Guo, Yonghong Yao, Jin Liu

**Affiliations:** ^1^ Department of Control Engineering Wuxi Institute of Technology Jiangsu 214000 China; ^2^ Laboratory of Advanced Design, Manufacturing & Reliability for MEMS/NEMS/OEDS School of Mechanical Engineering Jiangsu University Jiangsu 212013 China

**Keywords:** Adam, (Al−P)-ZnO, First-principles, Optoelectronic properties

## Abstract

The BP neural network optimized by the Adam algorithm was used to predict the defect formation energy of Al−P co‐doped ZnO systems with different concentrations of P replacing O under the presence of different concentrations of V_Zn_. It was found that the easily formed Al_Zn_Po‐1V_Zn_, Al_Zn_P_O_‐2V_Zn_, and Al_Zn_2P_O_‐1V_Zn_ systems. The first principles of density function were used to study the geometric, electronic, and optical properties of each system. The simulation results show that the bandgap values of the three systems have decreased relative to the intrinsic ZnO, among which Al_Zn_P_O_‐1V_Zn_ and Al_Zn_P_O_‐2V_Zn_ is still a p‐type conductive system, Al_Zn_P_O_‐2V_Zn_ has the highest conductivity. From the analysis of reflectivity, absorption rate, and light transmittance, Al_Zn_2P_O_‐1V_Zn_ has the most relatively excellent optical properties, followed by AlznPo‐2V_Zn_.

## Graphical abstract

The geometric structure, energy band structure, electronic state density and optical properties of Al−P co‐doped ZnO systems under the presence of different concentrations of V_Zn_ are investigated by using first‐principles.

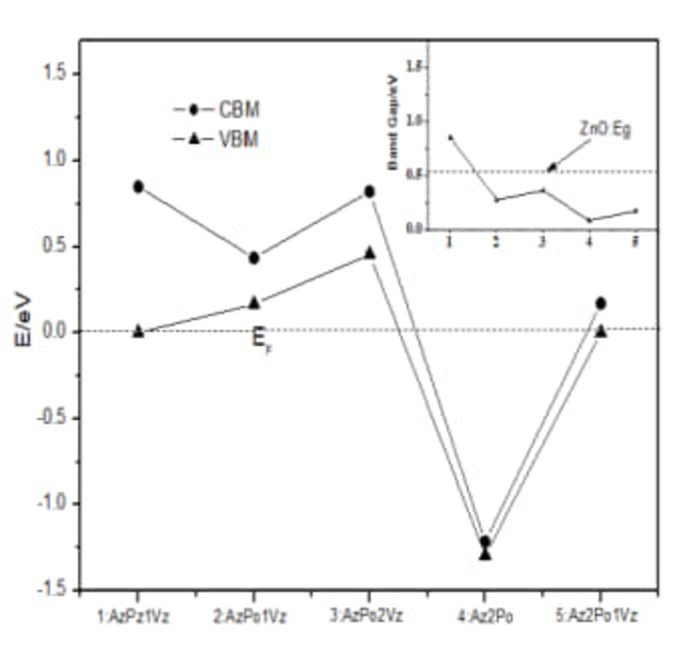

## 1 Introduction

ZnO is a direct bandgap semiconductor, which means its lowest CBM value and highest VBM value are at the same position, allowing for minimal energy consumption during electron transitions. Its exciton binding energy of up to 60 meV, excellent chemical and thermal stability, and low raw material cost are considered the most promising materials for the preparation of optoelectronic devices such as LEDs, transparent electrodes, and light‐emitting devices after gallium nitride.[^1^, ^2^, ^3^] The presence of donor defects such as oxygen vacancies (V_O_), zinc gaps (i_Zn_), and the incorporation of H atoms into the air during the natural growth of intrinsic ZnO can enhance n‐type conductivity.[^4^, ^5^, ^6^] However, n‐type ZnO can only partially meet the needs of the optoelectronic field. The preparation of repeatable and stable p‐type conductive ZnO thin films is a key factor in breaking through the bottleneck.[^7^, ^8^] The V‐group elements are considered one of the most promising dopants for p‐type ZnO, and P is one of them.[^9^, ^10^, ^11^, ^12^, ^13^, ^14^, ^15^] In recent years. Sharmila et al. prepared Al‐doped ZnO films using different methods and analyzed the structure and morphology characteristics of the films using XRD and SEM. They found that the photoconductivity of the doped films was significantly improved compared to the intrinsic ZnO.^[16]^ Wang et al. prepared Al−P co‐doped ZnO thin films on sapphire using RF magnetron sputtering and studied the effect of oxygen partial pressure on the properties of the films. It was found that the concentration of oxygen determines the concentration of donor and acceptor defects in the system, thereby affecting the conductivity type of the thin film.^[17]^ Considering the photoelectric characteristics of the V‐group P‐doped ZnO system and the influence of the main defect V_Zn_ on it, the defects can be compared and analyzed by various formation energies, and the calculation of the defect formation energies requires different model calculations, which is inefficient. Therefore, the algorithm is considered to improve the calculation efficiency.

The BP neural network obtains the error through the forward propagation of the activation function, compares it with the target value, and then reversely propagates to update the weight threshold. Repeat this process to obtain the optimal weight threshold for the training accuracy.^[18]^ The basic principle of the Adam method is to combine Momentum and RMSprop to further modify the weight and threshold of the BP algorithm to ensure a more reasonable prediction accuracy after training.^[19]^ Some scholars use ANN to predict the properties of relevant materials, which also brings enlightenment to this paper.[^20^, ^21^, ^22^] Therefore, the paper uses the BP neural network optimized by the Adam algorithm to train the defect stroke energy of each doped system and analyze the easily formed system. Then, the electronic structure and optical properties of Al−P co‐doped ZnO in the presence of V_Zn_ are calculated by the first principle to find out the doped system with excellent performance and can be implemented in the experiment, and the mechanism of related performance of Al−P co‐doped ZnO is theoretically disclosed.

## 2 Crystal Cell System, Network Model and Calculation Methods

### 2.1 Crystal Cell Model

**Figure 1:**
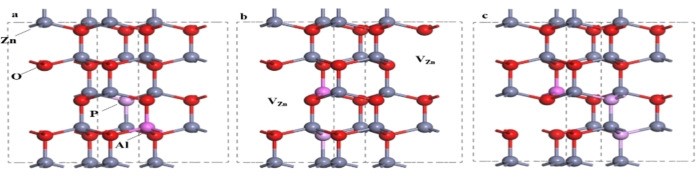
Unit cell model of each doping system: (a) Al_Zn_P_O_‐1V_Zn_; (b) Al_Zn_P_O_‐2V_Zn_;(c) Al_Zn_2P_O_‐1V_Zn_.

The stable phase of ZnO is of hexagonal gibbsite structure at room temperature and normal pressure. Therefore, based on the cell of ZnO, the model calculated in this paper expands one unit in the direction of a, b and c to obtain 2×2×2 supercells, including 16 ZnO, as shown in Figure 1. The three models are AlznPo‐1V_Zn_, AlznPo‐2V_Zn_ and Alzn2Po‐V_Zn_, respectively. Where Al_Zn_ represents one Al atom replacing one Zn, P_O_ represents one P atom replacing one O, 1V_Zn_ represents one Zn vacancy, and the preceding number represents the number of replacement atoms or vacancies. The doping position is selected by comparing the literature reference and the actual calculated energy.^[23]^

### 2.2 Formation Energy Predicted by the Network Model

**Figure 2:**
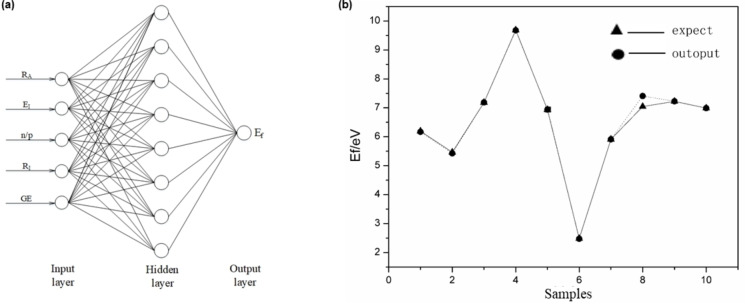
(a) Neural network structure; (b) Neural network prediction.

The formation energy of ZnO material system is an important parameter, which can reflect whether the material system is easy to form and stable under certain conditions.^[24]^ In this paper, the BP neural network model optimized by Adam is used to establish a three‐layer structure, and the model structure is shown in Figure 2(a). The first layer is the input layer. After using the correlation analysis of SPSS, five nodes of the input layer are determined as the atomic radius, ionization energy, valence radius, neutron‐proton ratio and environmental conditions. The second layer is the hidden layer that processes input node data. The third layer is the output layer, which is the key parameter of defect formation energy.

The activation function used in the network propagation process is the nonlinear activation function sigmoid. The Adam algorithm corrects the deviation value by using Momentum and RMSprop to the weight threshold value of different layers and then updates it. Figure 2(b) shows the comparison diagram of 10 groups of formation energy data predicted by the BP neural network structure optimized by the Adam algorithm after 140 groups of data training and the calculated formation energy data. It can be seen from the figure that the comparison error between the actual output result and the expected ideal result is very small, which proves the feasibility of this neural network prediction defect formation energy.

The formation energies of four kinds of defects in the Al−P co‐doped ZnO System with different concentrations of P replacing o are predicted by the neural network structure model, as shown in Table 1.

**Table 1:** ANN predicted the formation energy value of each system.

Number	System type	$E_{f}\left(ANN\right)$ (eV)
1	Al_zn_P_o_‐1V_zn_	−5.386
2	Al_zn_P_o_‐2V_zn_	−3.729
3	Al_zn_2P_o_‐1V_zn_	−5.112
4	Al_zn_2P_o_‐2V_zn_	8.747

It can be seen from the value of formation energy that the formation energy of the Al_Zn_2P_O_‐2V_Zn_ system is as high as 8.7 eV, which means that unless the growth conditions are strictly controlled, the system is difficult to occur naturally in doping, so this system is not considered for the time being. Other systems are relatively easy to form and suitable for modeling and first principles analysis.

### 2.3 Calculation Methods

In this study, the first principle of DFT calculation of plane wave pseudopotential is used, and PBE‐GGA is used to deal with the exchange‐correlation effect and periodic boundary conditions. This calculation is implemented using the Castep module in Material Studio. Due to the large number of atoms in the supercell model, the structural geometry of the model is optimized before calculation. The maximum atomic interaction force is set at 0.05 ev/Å, the maximum stress is set at 0.1 GPa, the maximum displacement is set at 0.002 Å, the convergence accuracy energy is set at 2.0×10^−5^ev, and the k‐point sampling in the Brillouin zone is set at 3×3×2. The calculation can be carried out after reaching the convergence conditions. The valence electrons involved in the calculation are Al:3 s^2^3p; P:3 s^2^3p^3^; Zn:3d^10^4 s^2^; O:2 s^2^2p^4^.

## 3 Result and Discussion

### 3.1 Structural Properties

Table 2 shows the lattice constants and volume changes of each system after geometric optimization. It can be seen from Table 2 that the length of the a‐axis of Al_Zn_P_O_‐1V_Zn_ is relatively reduced, while the length of the c‐axis is relatively increased, and the optimized cell volume is slightly reduced. Combined with the formation, it can be seen that the stability of this system is sufficient, so it is mainly the loss of Zn ions that leads to the slightly reduced volume of the system. The volume of the Al_Zn_P_O_‐2V_Zn_ system is much larger than that of the intrinsic system. Because the formation energy of this system is larger than that of the Al_Zn_P_O_‐1V_Zn_ system, and the distortion of the system is also larger, the number of doping changes between systems is the same, but its volume changes and the changes of a and c axes are larger; The formation energy of Al_Zn_2P_O_‐1V_Zn_ system is relatively small, but it is doped with more p atoms, so the volume change is greater.

**Table 2:** The lattice constant of each system.

system	a/nm	c/nm	c/a	V/nm^3^
ZnO	0.333	0.5378	1.615	0.414
Al_zn_P_o_‐1V_zn_	0.327	0.542	1.657	0.411
Al_zn_P_o_‐2V_zn_	0.347	0.529	1.524	0.421
Al_zn_2P_o_‐V_zn_	0.336	0.54	1.607	0.422

### 3.2 Analysis of Band Structure and Electronic Density of States

**Figure 3:**
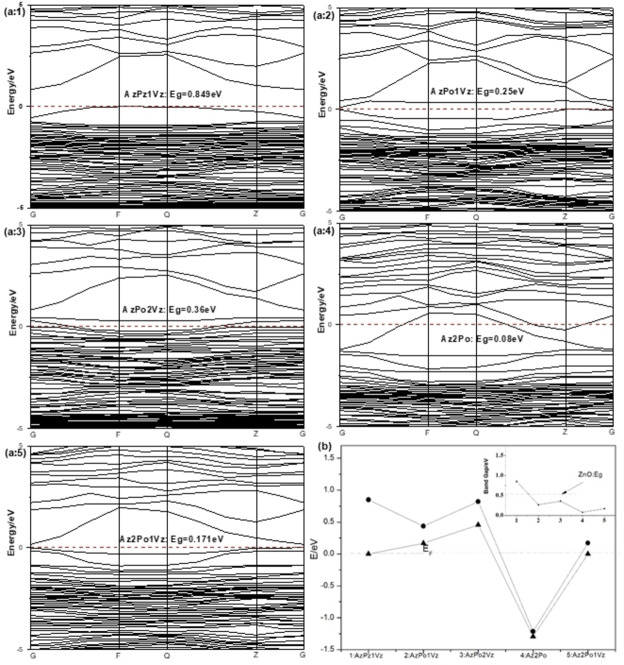
Energy level and band gap changes of different doping systems.

Figure 3(a) shows the energy band structure of the Al−P co‐doped ZnO system with P replacing O in the presence of V_Zn_, in which a:1 to a:5 represent the five band structures of Al_Zn_P_Zn_‐1V_Zn_, Al_Zn_P_O_‐1V_Zn_, Al_Zn_P_O_‐2V_Zn_, Al_Zn_P_O_ and Al_Zn_2P_O_‐1V_Zn_ respectively. Figure 3(a:2) is the energy band of Al_Zn_P_O_‐1V_Zn_ system. It is found that the energy level at the bottom of the conduction band becomes wider, and many new acceptor impurities appear at the top of the valence band. The two impurity levels change the energy band structure of the system, making the Fermi level enter the valence band. The system is p‐type conductive, and the band gap value is greatly reduced, and the carrier concentration becomes higher. Compared with the energy band of Al_Zn_P_Zn_‐1V_Zn_, it is found that the system becomes p‐type conductive more easily when P replaces O atoms. Figure 3(a:3) is the energy band of Al_Zn_P_O_‐2V_Zn_ system. It is found that the Fermi level of the system is deeper into the valence band. The p‐type conductivity is more obvious, which confirms that V_Zn_ is an acceptor doping, and the band gap value of the system is smaller than that of the intrinsic system, which is consistent with the literature analysis.^[25]^ Figure 3(a:5) represents the energy band of the Al_Zn_2P_O_‐1V_Zn_ system, which increases the doping concentration of P compared to the Al_Zn_2P_O_ system. The valence band and conduction band both shift upwards, and the Fermi level enters the bandgap region. The system exhibits a non‐degenerate state. From Figure 3(b), it can be concluded that the Al_Zn_P_O_‐1V_Zn_ and Al_Zn_P_O_‐2V_Zn_ systems are more suitable for preparing p‐type conductive transparent oxide thin films when analyzing the three easily formed doping systems. Combined with the Al_Zn_P_O_‐1V_Zn_ system, it is inferred that P_O_ doping introduces shallow donor impurities, which have a relatively greater impact than P_Zn_. Analyzing three easily formed doping systems, the Al_Zn_P_O_‐1V_Zn_ and Al_Zn_P_O_‐2V_Zn_ systems are more suitable for preparing p‐type conductive transparent oxide films.

**Figure 4:**
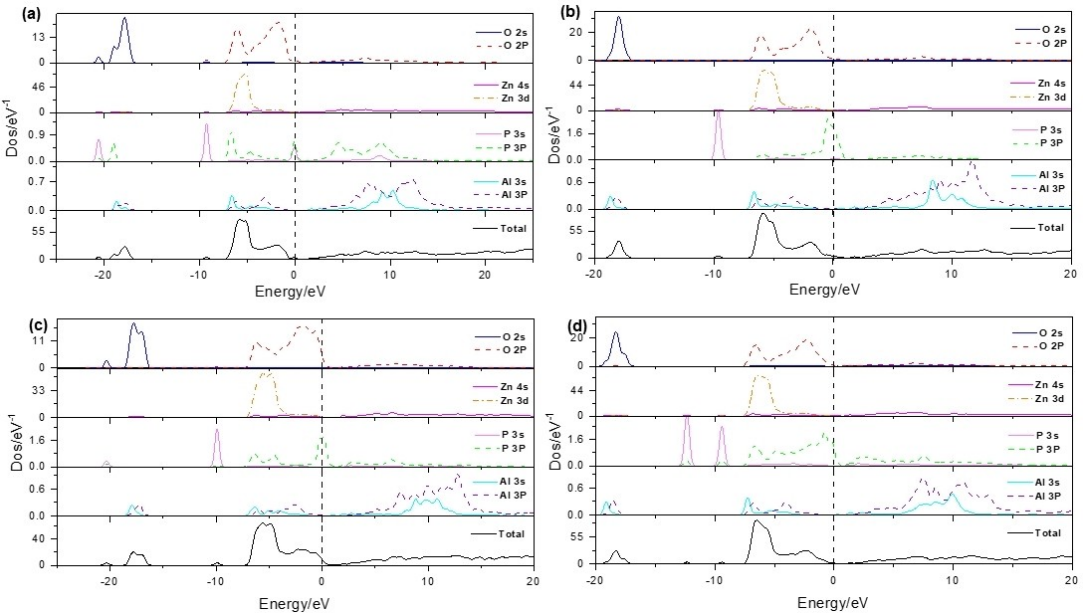
DOS and TDOS diagrams of various systems: (a) Al_Zn_P_Zn_‐1V_Zn_; (b) Al_Zn_P_O_‐1V_Zn_; (c) Al_Zn_P_O_‐2V_Zn_; (d) Al_Zn_2P_O_‐1V_Zn_.

Figure 4 shows the total density of States and the partial density of states of the four systems. Figure 4(b) shows the overall density of states (DOS) of the Al_Zn_P_O_‐1V_Zn_ system. Its conduction band is composed of Al‐sp orbital electrons, and its valence band is mainly composed of P‐sp, Zn‐3d and O‐2p orbital electrons. It is also found that P‐3p has a higher peak at the top of the valence band, which constitutes the top part of the valence band. At the same time, it is found that the Fermi level passes through the top of the valence band, and the system is p‐type conductive. Figure 4(c) shows the overall density of states of Al_Zn_P_O_‐2V_Zn_. It is observed that the DOS of the system moves towards the high‐energy region, and the Fermi level penetrates deeper into the valence band, indicating that the high concentration of the acceptor impurity V_Zn_ is dominant and the system is p‐type strengthened. Figure 4(d) shows the total density of states of Al_Zn_2P_O_‐1V_Zn_, which moves away from the Fermi level towards the lower energy region relative to b and c in the −10‐0 eV valence band range. The valence band electrons of O‐2p, Zn‐3d, and Al‐3p show the same trend, and the system is a non‐degenerate semiconductor. The results of state density analysis are consistent with the energy band diagram, and b and c systems are most suitable for preparing p‐type conductive oxide films.

**Figure 5:**
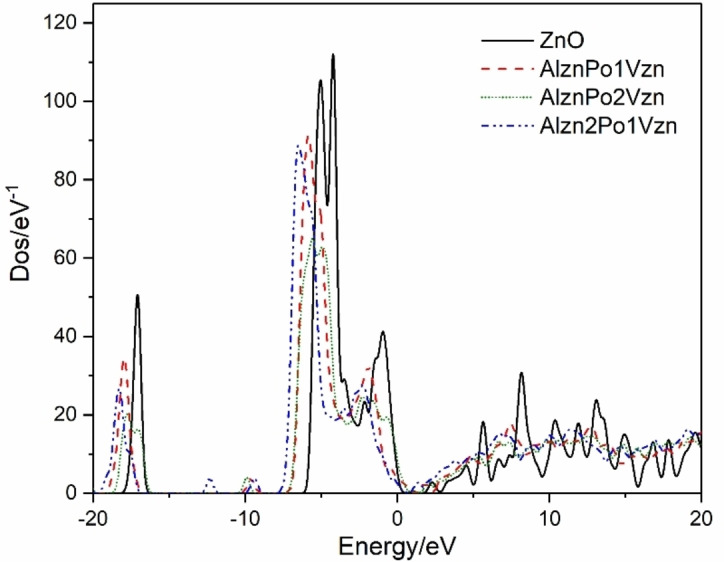
Comparison of DOS of various systems.

Figure 5 shows the DOS comparison diagram of the intrinsic system and Al−P co‐doped system with P replacing O in the presence of V_Zn_, analyzing P‐state orbital electrons and confirming the interaction between P and V_Zn_. Compared with the Al_Zn_P_Zn_ system, when one V_Zn_ appears, the P‐3p state density of the system moves to the high energy region, and the peak appears at the Fermi level, which indicates that the system begins to transform to the p‐type conductive system. By increasing the number of V_Zn_, it is found that the peak of P‐3p state density near the Fermi level disappears completely and becomes very discrete, but the state density is compact on the whole energy axis. The total energy of the density of the state is lower than that of the intrinsic system, and the presence of V_Zn_ makes the system close to the low‐energy region, and the DOS at 0–5 eV is closer to the Fermi level than the intrinsic system so that more conduction band electrons can pass through the Fermi level to form a p‐type conductive system. However, the dispersion of state density of the Al_Zn_2P_O_‐1V_Zn_ system at ‐ 5 to 0 eV is less than that of the other two systems, and it does not present p‐type conductivity.

**Figure 6:**
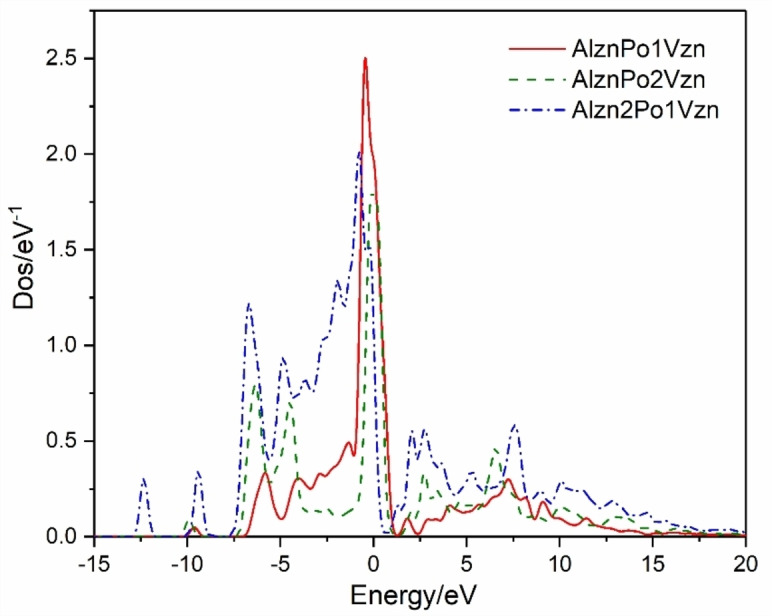
P‐3p partial‐wave state density of Vzn doped system with different concentrations.

Figure 6 shows the P‐3p partial density of states of different Zn vacancy doping systems. The specific effects of P and V_Zn_ on these three doped systems are determined by analyzing P‐state orbital electrons. By comparing the above two systems, it is found that with the increase of V_Zn_ concentration, the state density moves to the high energy region, and the peak crosses the Fermi energy level, the system shows p‐type conductivity; Increasing the doping concentration of P‐substituted O, it is found that the density of state moves back significantly, the peak near the Fermi level is on the left side of the Fermi level, and the system is non‐p type.

### 3.3 Conductivity Analysis

From the previous analysis of energy band and density of state, the systems with p‐type conductivity are Al_Zn_P_Zn_‐1V_Zn_ and Al_Zn_P_Zn_‐2V_Zn_, and their bandgap is better than the intrinsic bandgap. To further explore the change in system performance, it is necessary to calculate, analyze and compare the conductivity of these systems. The relative formula of conductivity is as follows:

(1) $$\sigma =\frac{nq^{2}\tau_{n}}{m^{*}}$$

Where n represents the electron concentration, and the change of the relative electron number can be obtained by integrating the electron density of the system from the conductivity Fermi level to the conduction band bottom in the total state density diagram. The electron concentrations of Al_Zn_P_O_‐1V_Zn,_ Al_Zn_P_O_‐2V_Zn_ and Al_Zn_2P_O_‐1V_Zn_ for the three systems are n1=1.36, n2=2.71 and n3=0.37, respectively.

q represents the electric quantity. The relative electric quantity of the four systems is roughly the same and is not included in the calculation.

τ represents the average free time. The calculated doping concentration of the system is greater than 1018 cm^−3^, which is the high doping concentration. Therefore, the average free time of each system is:

(2) $$\tau =N^{-1}T^{3/2}$$

In the formula, N represents the concentration of ionized particles, T is the temperature, and the average free time is inversely proportional to the impurity concentration. Therefore, based on the concentration of the doping system, τ1:τ2:τ3:τ4=31/2 : 15 : 31/3 is calculated.

m* is the effective mass of the electron, according to the formula:

(3) $$m^{*}=\hbar^{2}{\left(\frac{d^{2}E}{dk^{2}}\right)}^{-1}$$

Calculate the relative effective masses m*1, m*2, and m*3 for the three systems as 15.13, 15.77, and 13.07, respectively. The ratio obtained by comparing the conductivity of the four systems is as follows:
(4)

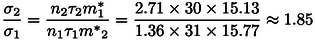




By comparing the relative conductivity ratios of the three systems, it can be concluded that in the Al−P co‐doped system where P replaces O in the presence of V_Zn_, the Al_Zn_P_O_‐2V_Zn_ system is suitable for preparing high‐performance p‐type conductive films based on energy band, density of states, and conductivity analysis.

### 3.4 Optical Properties

**Figure 7:**
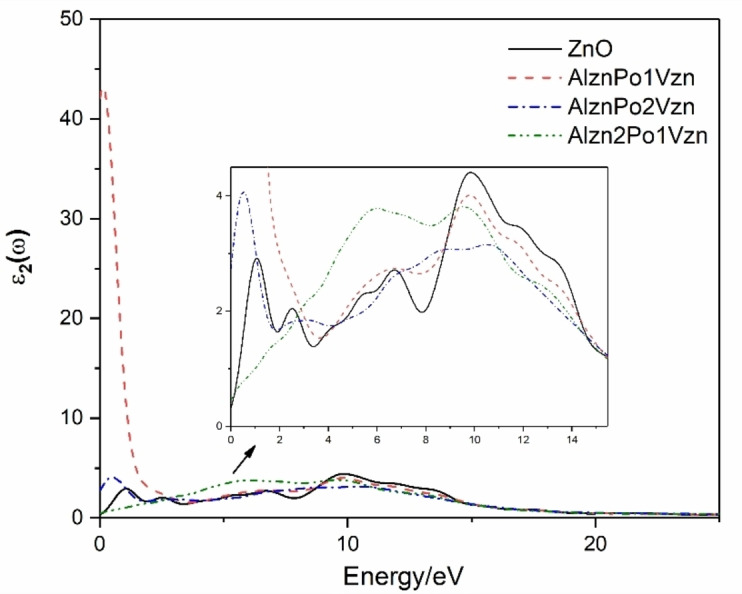
Imaginary part ϵ2 (ω) of the dielectric function of various systems.

Figure 7 represents the imaginary part ϵ 2 of the dielectric function of three easy‐to‐form co‐doped systems and intrinsic systems for P replacing O in the presence of V_Zn_. It can be seen from the figure that the peaks of the imaginary part diagram of the intrinsic ZnO system appear at the energies of 1.08 eV, 2.54 eV, 6.84 eV and 9.89 eV respectively. The first two peaks are from the electronic transition from the O‐2p state above the valence band to the Zn‐4 s state, the third peak is from the electronic transition from the Zn‐3d state below the valence band to the O‐2 s state, and the fourth peak is from the formation of the O‐2p state in the Zn‐3d state guide band below the valence band. For each doped system, the dielectric peak transits to the low energy region, and the whole dielectric peak shifts redly and gradually increases, resulting in a decrease of the band gap of the doped system, which is consistent with the analysis results of the band structure. At the same time, with the increase of Zn vacancy concentration, the dielectric peak moves to the long wave direction, so that the absorption of ZnO in the visible light region is enhanced. This may be caused by the interaction between Zn vacancy and ZnO electron not only for Al−P doped system with Zn vacancy but also P‐3 s in P‐element, which affects the coupling of electron wave function and O‐2p.

**Figure 8:**
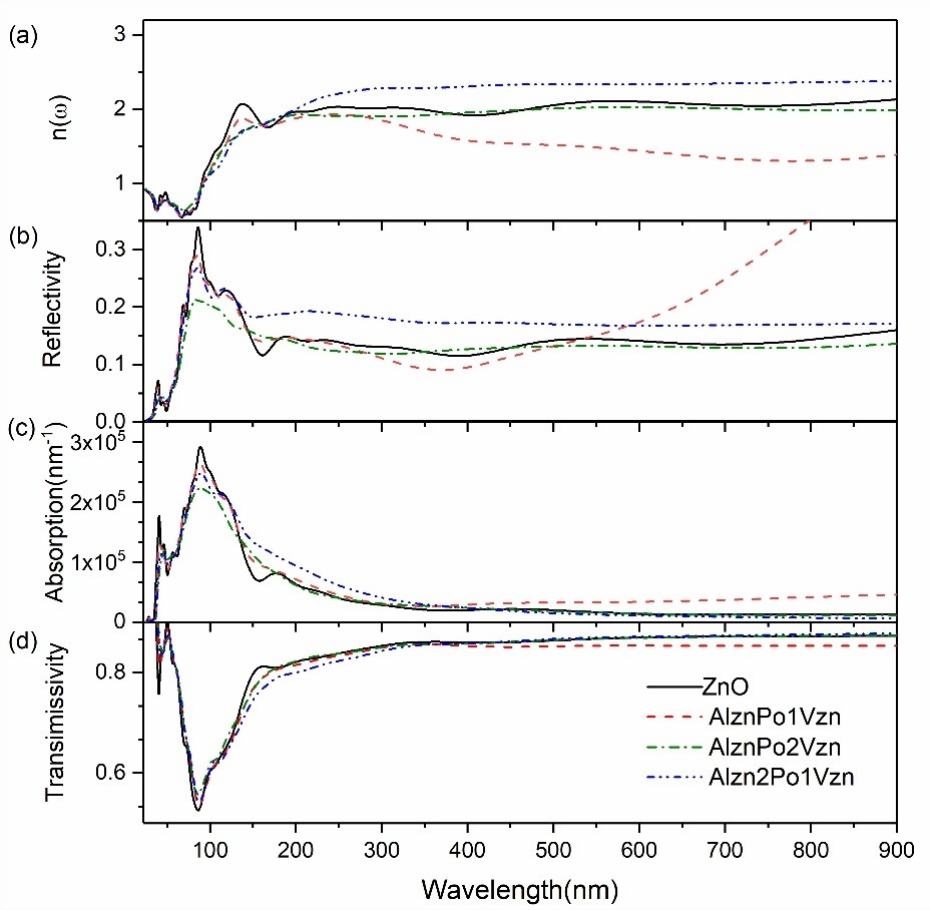
Optical spectrum: (a) refractive index; (b) reflectivity spectrum; (c) absorption spectrum; (d) transmittance spectrum.

Figure 8(a) represents the optical absorption spectra of the intrinsic system and AlznPo‐1V_Zn_, AlznPo‐2V_Zn_ and Alzn2Po‐1V_Zn_ systems. From the figure, it can be seen that the refractive index of Alzn2Po‐1V_Zn_ is the largest, the refractive index of AlznPo‐1V_Zn_ is the smallest, and the refractive index of AlznPo‐2V_Zn_ is very close to the refractive index of intrinsic ZnO, within the wavelength range of 170 nm–900 nm.

Figure 8(b) represents the optical reflectivity of three doping and intrinsic systems that are easily formed when P replaces O in the presence of V_Zn_. From the graph, it can be seen that the Al_Zn_2P_O_‐1V_Zn_ system has the worst reflectivity performance before entering the visible light wavelength range, and the AlZnPO‐1V_Zn_ system has the best performance. However, the change in reflectivity of the Al_Zn_P_O_‐1V_Zn_ system is relatively significant compared to other systems, with a sudden increase starting from 350 nm and continuing beyond the visible light wavelength range. The change is relatively abnormal, and its feasibility will be comprehensively analyzed based on its absorption and transmittance. The reflectivity of the AlznPo‐2Vzn system is superior to that of the intrinsic system except in the wavelength range of 400 nm.

Figure 8(c) represents a comparison of the light absorption rates of the intrinsic system and Al_Zn_P_O_‐1V_Zn_, Al_Zn_P_O_‐2V_Zn_, and Al_Zn_2P_O_‐V_Zn_. It can be seen that the absorption rates of the four systems have the same trend in the ultraviolet wavelength range. After entering the visible light range, the light absorption rate of the Al_Zn_P_O_‐1V_Zn_ system also suddenly increases and remains at the highest absorption rate position, which is consistent with the expression of reflectivity.

Figure 8(d) shows the variation of light transmittance for these four systems. Previously, it was analyzed that the reflectivity and absorption of the Al_Zn_P_O_‐1V_Zn_ system vary differently in the visible light range. In addition, this graph also shows that the light transmittance of this system sharply decreases after entering 380 nm, and the light transmittance has already decreased to below 85 %. Overall, although the Al_Zn_P_O_‐1V_Zn_ system is a p‐type conductive system with a small bandgap, its optical properties are poor and not suitable for experimental research and preparation. The light transmittance and absorption performance of the other three systems are almost the same, and the light transmittance in the visible light region is above 85 %. After comprehensive analysis, Al_Zn_P_O_‐2V_Zn_ is worth studying the Al_Zn_2P_O_‐1V_Zn_ system.

## 4 Conclusions

In this paper, the BP neural network optimized by the Adam algorithm is used to predict the defect formation energy of the Al−P co‐doped ZnO system under different concentrations of V_Zn_. The formation energy of each system is analyzed to obtain three more stable systems, namely Al_Zn_P_O_‐1V_Zn_, Al_Zn_P_O_‐2V_Zn_ and Al_Zn_2P_O_‐1V_Zn_ systems. Then, the first principle ultra‐soft pseudopotential method of density functional theory is used to study the geometry, band structure, electronic density of state, conductivity and optical properties of each system. The calculation results show that among the three kinds of easily formed doping systems, The Fermi levels of Al_Zn_P_O_‐1V_Zn_ and Al_Zn_P_O_‐2V_Zn_ systems entered the valence band, and the system showed p‐type conduction, and it was confirmed that V_Zn_ was acceptor doping. Al_Zn_P_O_‐1V_Zn_ system is a p‐type conductive system with a small bandgap, but its optical property is very poor, so it is not suitable for experimental study and preparation. The light transmittance of the visible light region of Al_Zn_P_O_‐2V_Zn_ system is more than 86 %. This system is suitable for preparing P‐type conductive oxide thin films with good performance.

## Conflict of Interests

The authors declare no conflict of interest.

5

## Data Availability

The data that support the findings of this study are available from the corresponding author upon reasonable request.
